# Social and environmental malaria risk factors in urban areas of Ouagadougou, Burkina Faso

**DOI:** 10.1186/1475-2875-8-13

**Published:** 2009-01-13

**Authors:** Meili Baragatti, Florence Fournet, Marie-Claire Henry, Serge Assi, Herman Ouedraogo, Christophe Rogier, Gérard Salem

**Affiliations:** 1 Parasite Biology and Epidemiology Research Dept, UMR 6236 – URMITE, IMTSSA, Parc du Pharo, BP46, 13998 Marseille-Armées, France; 2 Environnement urbain et transition sanitaire en Afrique de l'Ouest, IRD, 01 BP 182, Ouagadougou, Burkina Faso; 3 Centre Muraz, BP 360 Bobo-Dioulasso, Burkina-Faso; 4 Institut Pierre Richet/Institut National de Santé Publique, BP V 47 Abidjan, Côte d'Ivoire; 5 Institut de Recherche en Sciences de la Santé (IRSS/CNRST) – 03 BP 7192 Ouagadougou, Burkina Faso; 6 Laboratoire Espace, Santé et Territoire, Université Paris X-Nanterre, 200 avenue de la République, 92001 Nanterre Cedex, France

## Abstract

**Background:**

Despite low endemicity, malaria remains a major health problem in urban areas where a high proportion of fevers are presumptively treated using anti-malarial drugs. Low acquired malaria immunity, behaviour of city-dwellers, access to health care and preventive interventions, and heterogenic suitability of urban ecosystems for malaria transmission contribute to the complexity of the malaria epidemiology in urban areas.

**Methods:**

The study was designed to identify the determinants of malaria transmission estimated by the prevalence of anti-circumsporozoite (CSP) antibodies, the prevalence and density of *Plasmodium falciparum *infection, and the prevalence of malarial disease in areas of Ouagadougou, Burkina-Faso. Thick blood smears, dried blood spots and clinical status have been collected from 3,354 randomly chosen children aged 6 months to 12 years using two cross-sectional surveys (during the dry and rainy seasons) in eight areas from four ecological strata defined according to building density and land tenure (regular versus irregular). Demographic characteristics, socio-economic information, and sanitary and environmental data concerning the children or their households were simultaneously collected. Dependent variables were analysed using mixed multivariable models with random effects, taking into account the clustering of participants within compounds and areas.

**Results:**

Overall prevalences of CSP-antibodies and *P. falciparum *infections were 7.7% and 16.6% during the dry season, and 12.4% and 26.1% during the rainy season, respectively, with significant differences according to ecological strata. Malaria risk was significantly higher among children who i) lived in households with lower economic or education levels, iii) near the hydrographic network, iv) in sparsely built-up areas, v) in irregularly built areas, vi) who did not use a bed net, vii) were sampled during the rainy season or ii) had traveled outside of Ouagadougou.

**Conclusion:**

Malaria control should be focused in areas which are irregularly or sparsely built-up or near the hydrographic network. Furthermore, urban children would benefit from preventive interventions (e.g. anti-vectorial devices or chemoprophylaxis) aimed at reducing malaria risk during and after travel in rural areas.

## Background

According to the UN Population Fund's State of World Population 2007 report, Africa had an urbanization level of 38% in 2005, and 72% of sub-Saharan Africa's urban population lived in slum conditions. By 2040, over half of the population of Africa is expected to live in urban areas. It has been shown that the level of malaria endemicity in sub-Saharan Africa is generally lower in these areas than in rural areas [1]. It is generally considered that suitable vector breeding sites are scarce in highly populated areas despite evidences of the adaptation of malaria vectors to African urban environments [2,3]. Despite low endemicity, a high proportion of fevers are presumptively treated as malaria in urban areas and the anti-malarial drug consumption is higher than in rural areas [4,5]. In such context, misdiagnosis of malaria could favour the selection and the spread of drug resistance [4] and contributes to increasing ill-health due to delayed diagnosis of non-malaria diseases, overburdened health services and increased cost to patient and to health facilities [6]. As a result of the low endemicity, the acquisition of semi-immunity is delayed among children and adults [7,8]. Then, they are exposed to more severe malaria than those living in rural areas.

Moreover there could be major heterogeneities in malaria transmission [1,9-18] and in other malariometric indices [16,19-27] between different areas of a town and between cities, combined with major differences in access to health structures and cares. Because of these heterogeneities, it is necessary to target the malaria control interventions to specific urban populations and areas on which we need more detailed information. In this perspective, remote sensing is increasingly considered as a cost-effective solution to monitoring urbanization, targeting malaria control interventions or estimating malaria burden in urban areas [28-30]. The nature of urban environment may also make easier the malaria control because the high population density, the social and economical urban context and the focused nature of malaria vectors breeding sites facilitate increased coverage of interventions, improved access to preventative and curative measures and then higher impact of integrated malaria control strategies [16,28,31,32]. However, most of malaria research have been done in rural areas and the strategies including vector control and the diagnosis and treatment of infection should be tailored to the urban context [16,33]. Therefore, there is an urgent need for exploring the malaria risk factors in urban settings [1,33,34].

The aim of the present study is to identify the determinants of malaria transmission, the prevalence and density of *Plasmodium falciparum *infection, and the prevalence of malaria disease, in different areas of Ouagadougou.

Previous studies have investigated the prevalence of malaria in Ouagadougou, Burkina Faso. A study conducted by Sabatinelli *et al *[35] in 0 to 5 year-old children during the peak of the 1984 transmission season (August-September) showed an overall parasite prevalence of 16% and significant differences between areas of the town. Dabire [36] also reported different prevalence rates between town center, areas across the canals and areas near artificial lakes. Wang *et al *[25] showed that parasitaemia prevalence was relatively high (48.3%) in school children during the cold and dry season of 2002, and that there was a gradient of endemicity between the urban center and the periphery of Ouagadougou. The results of the present study complete the picture of malaria epidemiology in Ouagadougou, enable to identify at-risk populations, and consequently to have some basis for planning and focusing malaria control measures in Ouagadougou in particular, and in Africa's urban areas in general.

## Materials and methods

### Study area and design

Ouagadougou is the capital of Burkina Faso (lat. 12°22'N and long. 1°31'W), and had a population of around 1,086,000 inhabitants in 2006. The annual rainfall is 750 to 900 mm. The rainy season is between June and October, the cold and dry season is between November and January, and the hot and dry season is between February and May. Three artificial lakes are located within the city intended to supply water to the population.

There is a variety of ecological situations and urbanization processes in Ouagadougou. The first step in the present study was to identify these different situations and processes. A two-stage stratified sampling approach was used. The stratification was carried out using a SPOT 5 panchromatic satellite image of Ouagadougou along with cadastral data. These data were completed and validated by field observations. ERDAS software was used for analysis of the satellite image, and ArcView software was used to compute building density level.

The first stratification criterion was the allotment status which opposed regular to irregular areas. The regular city is characterized by a network of hierarchical streets, and the presence of basic services like electricity, running water, health structures and schools. In the regular area, land is allotted by administration. The irregular areas are composed of spatially-disorganized districts, that lack services. In the irregular city, land is given from informal delivery systems. The regularity of the area was chosen as a marker of vulnerability of the population, habitants of the irregular city being more vulnerable. The second stratification criterion was the building density considered as a proxy of the population density that could be a risk factor for infectious diseases and malaria transmission. The density of building was calculated at a cadastral unit scale called sections (survey areas were constituted by a pool of sections having the same building density). The units have been categorized in non built-up, sparsely built-up and densely built-up units, according to the density of building. Non buit-up units were discarded from the sampling.

**Figure 1:**
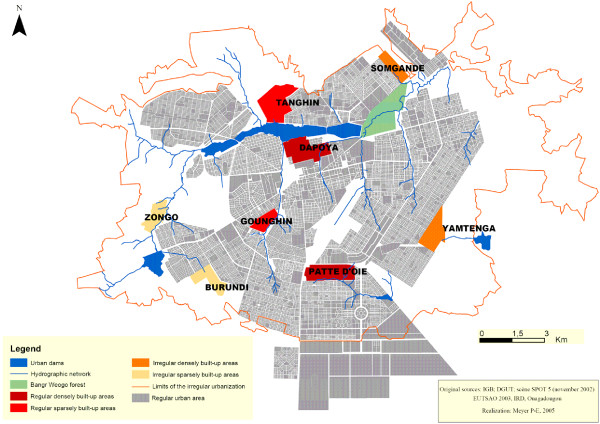
**Map of Ouagadougou including study areas, hydrographic network and dams, regular urban areas and limits of the irregular urbanization in 2004**.

Therefore, four strata representative of four different ecological situations in Ouagadougou were defined according to the regularity and density criteria. In each stratum, two survey areas were selected for the study: two densely built-up irregular areas (Somgande and Yamtenga), two sparsely built-up irregular areas (Burundi and Zongo), two densely built-up regular areas (Dapoya and Patte d'oie) and two sparsely built-up regular areas (Gounghin and Tanghin) (Figure 1). In regular areas, a probabilistic selection of parcels was made using cadastral data and a set of randomly generated numbers. In irregular areas, randomly selected pairs of geographic coordinates (X, Y) were marked on aerial photographs and if these coordinates corresponded to a compound, it was used as the starting point of a path.

To ensure a sufficient exposure to urban lifestyle, only households whose head had at least five years of residence in Ouagadougou were eligible. Consequently, in each selected yard, the household was eligible only if the head of household was born in Ouagadougou or had been living in Ouagadougou for more than five years, as we supposed that anyone having lived five years in a town would have acquired a urban way of life. In an eligible household, all children aged 6 months to 12 years and adults aged over 35 years were invited to participate in the study.

The health determinants studied were the following: anaemia, malaria and dengue fever in children, nutritional status in children and adults, dental status in children up to 6 years old and adults, hypertension in adults. This paper focuses only on malaria in children between 6 months and 12 years of age.

### Data collection and laboratory examination

Two cross-sectional surveys were implemented to take seasonal variations in health into account (April-May and September-October 2004). A mobile health post was established in each area at the time of the survey, with a team including two sociologists to interview participants, and a medical doctor for basic clinical examination and blood sampling. A questionnaire was administered to mothers (or carers) on socio-economic information (income, equipment of the household), demographic characteristics (age, gender, duration of residence in Ouagadougou, education of the head of the household and travel of the children outside of Ouagadougou), and sanitary data (malaria prevention and date of the most recent malaria for the children). Environmental data – geographic coordinates of the compound, distance from the compound to the closest drinking fountain, hydrographic network or artificial lake, and urban variables (land tenure and building density of the area) – were also recorded for each compound. Each child was clinically examined and his/her axillary temperature was measured. A temperature ≥ 37.5°C was considered to be a fever. Blood samples were collected by fingerprick, both for thick blood smears and spotting on filter paper. The team was trained prior to data collection, and then supervised by the multidisciplinary research team composed of epidemiologists and medical geography specialists. Sick children were treated free-of-charge according to the clinical diagnosis made by the medical doctor.

The research protocol was validated by the Ethics Committee for Research in Health of Burkina Faso. The mother, the father or the legal guardian of each child formally agreed that he/she takes part in the investigation after being well informed of the study and before his/her inclusion in the study.

Thick blood smears were Giemsa-stained in the field and examined at the Centre Muraz in Bobo-Dioulasso to identify *Plasmodium *species. *Plasmodium falciparum *was the predominant species found. Asexual stages of *P. falciparum *were counted in the blood volume occupied by 200 leukocytes and parasite density was calculated by assuming 8,000 leukocytes/μL of blood. Thick blood smears from each area were read by the same experienced technician, under the supervision of a parasitologist. The technicians were also compared on the same set of blood samples. The rate of parasite detection and parasite density estimates did not differ significantly between technicians. Cross-check quality control was regularly conducted on a randomly selected sample representing 10% of all thick smears. Blood samples for detection of anti-circumsporozoite antibodies (anti-CSP antibodies) were collected on filter paper (No 1; Whatman International Ltd., Maidstone, UK). Blood spots were dried and stored with silica gel at ambient temperature. Antibodies (IgG) to the *P. falciparum *circumsporozoïte protein were detected by the ELISA test by using a technique described elsewhere [37,38] at the Institute for Tropical Medicine of the French Army in Marseille, France.

### Statistical analysis

Four dependent variables were analysed: i) the prevalence of *P. falciparum *anti-CSP antibodies representing exposure to malaria transmission [4,37,39,40], ii) the prevalence of *P. falciparum *infection, iii) *P. falciparum *parasite density (only in positive children, as a continuous variable by taking the log of the density) and iv) the prevalence of *P. falciparum *malaria attacks. Two definitions were used for a malaria attack: presence of fever with a parasitaemia > 1,000 *P. falciparum *asexual stages/μL of blood (definition A), and presence of fever with a parasitaemia > 0 (definition B). To analyse these four dependent variables, socio-economic, demographic, sanitary and environmental information concerning the children or their families and the households or the compounds was used. The age variable was broken into two classes of equivalent sizes: 0–6 years and 7–12 years. An equipment index and an education level index were calculated for each family. The equipment index, determined by possession of assets, is a proxy for socio-economic status. Telephone (mobile or not), television, refrigerator, ventilator, bed, living room, and motorcycle were considered in the same component. Two categories, high and low equipment index, were determined. Education level was estimated by whether the head of the household had attended school, whatever the duration.

First, a descriptive analysis of the dependent and independent variables was carried out, taking into account the design of the study for the calculation of confidence intervals for dependent variables. The spatial distribution of the dependent variables in each area was also analysed. The index proposed by Moran was used to test for spatial autocorrelations [41]. Moran's index tested if measured variables from neighbouring compounds tended to be more similar than those from arbitrarily chosen compounds (1,000 random permutations of the compounds in the town were used). For qualitative variables, rates among children in the compounds were estimated by using empirical Bayes index to adjust the Moran's index taking into account the different numbers of children used to calculate the rates [41]. Neighbours of a compound were defined as the four closest compounds, and equal weights were attributed to each of these neighbors. Bivariate analyses were then performed, and all the variables with a p-value of less than 0.2 were entered into multivariate models. These models were fitted using a manual backward step-by-step procedure to select the significant variables. The urban variables (building density and land tenure) were forced into the model and relevant interactions were then tested. Significance was determined using likelihood ratio tests. Logistic regression models were employed for the binary indicators and linear regression models for parasite density. Since there were generally several individuals in each compound and several compounds in each area, the assumption of independence of observations did not necessarily hold. To take into account the clustering of participants within compounds and areas, mixed models were used with the clustering variables (i.e. the compound and the area) as nested random effects. The models were checked and the random-effects were tested for spatial correlation. In addition, concerning the binary indicators, the adequacy between predicted and observed probabilities of these indicators in each area was checked, and the ROC curve and the area under the curve were examined to evaluate the accuracy of the model. The statistical analysis was performed using the *R *software package, version 2.5.1, along with *lme4 *and *survey*. Significance was chosen as p-values less than 0.05.

## Results

### Study population

This study enrolled a total of 3,354 children, with at least 214 children in each selected area. Over the year of data collection, no seasonal fluctuation was observed in patient attendance. The number of children enrolled in each compound varied from one to 16, with a median of three children. Parasitaemia was detected in 728 children (22%), the presence of anti-CSP antibodies measuring the exposure to malaria transmission was detected in 343 children (10%), and 31 children (1%) were undergoing a clinical malaria attack during the data collection. The mean of the parasitaemia among positive children was 602 *P. falciparum *asexual parasites/μL of blood (95%CI: 545–665). The number of children, households and compounds enrolled in the study, the dependent variables, the qualitative independent variables reported as potential risk factors are shown in Table 1. The quantitative independent variables are summarized in Additional file 1. The prevalence of bed net use varied from 14.4% to 35.2% according to areas (Table 1), and only 15% of these bed nets were impregnated by insecticides.

**Table 1:** Description of the areas.

Land tenure	Irregular				Regular			
Building density	Sparse		Dense		Sparse		Dense	
Area	Burundi	Zongo	Somgandé	Yamtenga	Gounghin	Tanghin	Dapoya	Patte d'oie
No. compounds	188	194	176	223	132	170	103	82
No. households	194	194	178	223	148	172	128	86
No. of children aged 6 months-12 years	497	474	430	549	397	445	348	214
No. of children examined during dry season	237	201	209	283	184	205	113	132
No. of children examined during rainy season	260	273	221	266	213	240	235	82
Presence of anti-CSP antibodies*	63	69	38	48	27	49	25	24
% (95%CI)	12.7 (9.8–15.6)	14.6 (11.4–17.7)	8.8 (6.2–11.5)	8.7 (6.4–11.1)	6.8 (4.3–9.3)	11 (8.1–13.9)	7.2 (4.5–9.9)	11.2 (7–15.4)
Presence of *P. falciparum *parasitaemia	121	153	126	92	46	127	43	20
% (95%CI)	24.3 (20.6–28.1)	32.3 (28.1–36.5)	29.3 (25–33.6)	16.8 (13.6–19.9)	11.6 (8.4–14.7)	28.5 (24.3–32.7)	12.4 (839-15.8)	9.3 (5.4–13.2)
*P. falciparum *parasite density among positive children (/mL) Geometric mean (95%CI)	545 (365–735)	735 (545–992)	545 (403–735)	602 (446–898)	898 (446–1808)	602 (446–898)	446 (270–735)	221 (122–365)
Age (%)								
0–6 y.	256 (51.5)	287 (60.5)	228 (53.0)	304 (55.4)	188 (47.4)	216 (48.5)	191 (54.9)	101 (47.2)
7–12 y.	241 (48.5)	187 (39.5)	202 (47.0)	245 (44.6)	209 (52.6)	229 (51.5)	157 (45.1)	113 (52.8)
Sex (%)								
Female	238 (47.9)	240 (50.6)	204 (47.4)	271 (49.4)	208 (52.4)	223 (50.1)	181 (52.0)	106 (49.5)
Male	259 (52.1)	234 (49.4)	226 (52.6)	278 (50.6)	189 (47.6)	222 (49.9)	167 (48.0)	108 (50.5)
Use of bed net (%)								
No	382 (76.9)	307 (64.8)	312 (72.6)	445 (81.1)	324 (81.6)	381 (85.6)	190 (54.6)	173 (80.8)
Yes	115 (23.1)	167 (35.2)	118 (27.4)	104 (18.9)	73 (18.4)	64 (14.4)	158 (45.4)	41 (19.2)
Equipment (%)								
Low	301 (60.6)	314 (66.2)	304 (70.7)	359 (65.4)	114 (28.7)	280 (62.9)	102 (29.3)	63 (29.4)
high	196 (39.4)	160 (33.8)	126 (29.3)	190 (34.6)	283 (71.3)	165 (37.1)	246 (70.7)	151 (70.6)
Education level (%)								
low	216 (43.5)	224 (47.3)	284 (66.1)	235 (42.8)	191 (48.1)	251 (56.4)	149 (42.8)	88 (41.1)
high	281 (56.5)	250 (52.7)	146 (33.9)	314 (57.2)	206 (51.9)	194 (43.6)	199 (57.2)	126 (58.9)
Travel outside of Ouagadougou the preceding month (%)								
no	455 (91.5)	448 (94.5)	409 (95.1)	518 (94.3)	367 (92.4)	425 (95.5)	310 (89.1)	192 (89.7)
yes	42 (8.5)	26 (5.5)	21 (4.9)	31 (5.7)	30 (7.6)	20 (4.5)	38 (10.9)	22 (10.3)
Distance from the compounds to the closest hydrographic network (%)								
< 200 m	32 (6.4)	305 (64.3)	250 (58.1)	0 (0)	92 (23.2)	20 (4.5)	87 (25)	89 (41.6)
200–500 m	169 (34.0)	162 (34.2)	180 (41.9)	91 (16.6)	233 (58.7)	94 (21.1)	133 (38.2)	107 (50.0)
> 500 m	296 (59.6)	7 (1.5)	0 (0)	458 (83.4)	72 (18.1)	331 (74.4)	128 (36.8)	18 (8.4)
Distance from the compounds to the closest drinking fountain (%)								
< 200 m	193 (38.8)	5 (1.1)	103 (24.0)	185 (33.7)	151 (38.0)	391 (87.9)	283 (81.3)	39 (18.2)
200–500 m	272 (54.7)	147 (31.0)	320 (74.4)	304 (55.4)	246 (62.0)	54 (12.1)	65 (18.7)	168 (78.5)
> 500 m	32 (6.5)	322 (67.9)	7 (1.6)	60 (10.9)	0 (0)	0 (0)	0 (0)	7 (3.3)
Distance from the compounds to the closest the closest artificial lake (%)								
< 200 m	0 (0)	0 (0)	0 (0)	0 (0)	0 (0)	0 (0)	33 (9.5)	0 (0)
200–500 m	0 (0)	0 (0)	0 (0)	0 (0)	0 (0)	78 (17.5)	126 (36.2)	0 (0)
> 500 m	497 (100)	474 (100)	430 (100)	549 (100)	397 (100)	367 (82.5)	189 (54.3)	214 (100)

* Anti circumsporozoite protein antibodies

### Prevalence of anti-CSP antibodies

Anti-CSP antibodies are considered to be a sero-epidemiological marker of exposure to malaria transmission. The age of child, whether the child had traveled out of Ouagadougou during the preceding month, the season, the building density of the living area and the distance of the compound to the hydrographic network were significantly associated with the prevalence of anti-CSP antibodies in bivariate analysis (Table 2). According to the multivariate random-effect logistic regression model (Table 3), the prevalence of anti-CSP antibodies was higher among the oldest children (OR = 2.6; 95%CI: 2.0–3.35) and among the children traveling outside of Ouagadougou (OR = 1.91; 95%CI: 1.26–2.9). It was lower when the compound was far from the hydrographic network (OR = 0.60; 95%CI: 0.43–0.84 for the category 200 m–500 m and 0.66; 95%CI: 0.48–0.9 for the category > 500 m). The effect of the use of a bed net was significant only during the rainy season (OR = 0.60; 95%CI: 0.40–0.88). The effect of the season was significant only for the children who did not use a bed net (OR = 2.06; 95%CI: 1.52–2.79). Concerning the urban variables, the prevalence of anti-CSP antibodies was higher when the living area was irregular (OR = 1.41; 95%CI: 1.08–1.86) and the building density was sparse (OR = 1.41; 95%CI: 1.08–1.83). The observed and expected prevalences in each area were also examined, and appeared quite similar. The area under the ROC curve was 0.80. Gender, equipment and education level, and the distance of the compound to the drinking fountain or artificial lakes were not significant variables for explaining the prevalence of anti-CSP antibodies.

**Table 2:** Bivariate analysis of the dependent variables.

	Prevalence of anti-CSP antibodies					Prevalence of infection				Parasite density (among positive children)			
Variable	Modality	% (95% CI)	OR	95% CI	p-value	% (95% CI)	OR	95% CI	p-value	Geometric mean (95% CI)	Multiplicative factor	95% CI	p-value
Age	0–6 y.	6.4 (5.3–7.6)	1			17.5 (15.7–19.3)	1			735 (602–898)	1		
	7–12 y.	14.5 (12.7–16.2)	2.57	2.01–3.28	< 0.001	26.4 (24.2–28.6)	1.92	1.61–2.29	< 0.001	545 (446–602)	x0.74	0.57–0.97	0.031
Sex	Female	10.3 (8.8–11.8)	1			22.3 (20.3–24.3)	1			602 (493–665)	1		
	Male	10.2 (8.7–11.6)	0.97	0.77–1.22	0.789	21.1 (19.1–23)	0.9	0.76–1.08	0.267	602 (493–735)	x1.06	0.81–1.39	0.673
Use of bed net	no	10.7 (9.5–11.9)	1			23.3 (21.6–24.9)	1			545 (493–665)	1		
	yes	8.8 (6.9–10.7)	0.77	0.58–1.03	0.074	17 (14.5–19.6)	0.62	0.49–0.78	< 0.001	735 (545–992)	x1.2	0.85–1.71	0.301
Land tenure	regular	8.9 (7.4–10.4)	1			16.8 (14.9–18.8)	1			602 (446–735)	1		
	irregular	11.2 (9.8–12.6)	1.29	0.95–1.77	0.142	25.2 (23.3–27.2)	2.07	1.1–3.88	0.043	602 (493–735)	x1.03	0.76–1.40	0.848
Building density	high	8.8 (7.3–10.2)	1			18.2 (16.3–20.2)	1			545 (403–665)	1		
	sparse	11.5 (10–12.9)	1.36	1.04–1.79	0.041	24.7 (22.7–26.6)	1.7	0.83–3.48	0.177	665 (545–812)	x1.23	0.92–1.66	0.167
Season	dry	7.7 (6.4–9.1)	1			16.6 (14.8–18.5)	1			270 (245–330)	1		
	rainy	12.4 (10.9–13.9)	1.72	1.35–2.2	< 0.001	26.1 (24.1–28.2)	1.94	1.59–2.37	< 0.001	898 (735–1097)	x3.28	2.48–4.34	< 0.001
Equipment	low	11 (9.6–12.4)	1			27.7 (25.6–29.7)	1			602 (545–735)	1		
	high	9.3 (7.8–10.8)	0.86	0.67–1.1	0.223	14.5 (12.7–16.3)	0.5	0.41–0.62	< 0.001	545 (403–665)	x0.8	0.58–1.09	0.163
Education level	low	10.9 (9.4–12.4)	1			25.6 (23.5–27.8)	1			602 (493–665)	1		
	high	9.6 (8.2–11)	0.85	0.67–1.08	0.191	17.9 (16.1–19.8)	0.63	0.52–0.77	< 0.001	602 (493–735)	x1.03	0.77–1.38	0.851
Travel out of Ouagadougou the preceding month	no	9.8 (8.8–10.8)	1			21.7 (20.3–23.1)	1			545 (493–665)	1		
	yes	16.1 (11.3–20.8)	1.83	1.24–2.69	0.004	21.7 (16.4–27.1)	1.11	0.78–1.58	0.593	1339 (735–2208)	x2.16	1.26–3.70	0.005
Distance to the closest hydrographic network	< 200 m	13 (10.8–15.3)	1			23.5 (20.7–26.4)	1			602 (18–22026)	1		
	200–500 m	8.5 (6.9–10.1)	0.62	0.46–0.86	0.013	21.6 (19.3–24)	1.12	0.86–1.47	0.71	493 (403–665)	x0.83	0.58–1.20	0.305
	> 500 m	9.9 (8.3–11.5)	0.72	0.51–1.01		20.5 (18.3–22.7)	1.07	0.76–1.51		665 (15–29733)	x1.08	0.76–1.55	
Distance to the closest drinking fountain	< 200 m	9.7 (8.1–11.3)	1			20.2 (18.1–22.4)	1			493 (403–602)	1		
	200–500 m	9.9 (8.4–11.4)	0.99	0.74–1.33	0.389	20 (18–22)	1.02	0.8–1.3	0.043	735 (602–898)	x1.42	1.01–1.97	0.122
	> 500 m	13.1 (9.9–16.3)	1.39	0.94–2.07		32.7 (28.3–37.2)	1.64	1.09–2.47		545 (20–16318)	x1.03	0.66–1.61	
Distance to the closest artificial lake	< 200 m	6.1 (0–14.2)	1			12.1 (1–23.3)	1			365 (40–3641)	1		
	200–500 m	10.3 (6.1–14.5)	1.63	0.32–8.33	0.81	23.5 (17.7–29.4)	1.49	0.4–5.58	0.206	493 (18–14765)	x1.4	0.19–10.31	0.742
	> 500 m	10.3 (9.2–11.3)	1.48	0.31–7.2		21.7 (20.2–23.1)	0.95	0.26–3.51		602 (545–665)	x1.67	0.25–11.35	

**Table 3:** Multivariate analysis of the prevalence of anti-circumsporozoite protein (CSP) antibodies.

	Prevalence of anti-CSP antibodies				
	Variable	Modality	OR	95% CI	p-values
	Age	0–6 y.	1		< 0.001
		7–12 y.	2.6	2.0–3.35	
	Land tenure	regular	1		0.025
		irregular	1.41	1.08–1.86	
	Building density	high	1		0.032
		sparse	1.41	1.08–1.83	
	Travel outside of	no	1		0.003
	Ouagadougou the preceding month	yes	1.91	1.26–2.90	
	Distance to hydrographic network	< 200 m	1		0.006
		200–500 m	0.6	0.43–0.84	
		500 m	0.66	0.48–0.90	
Dry season	Use of bed net	no	1		0.29
		yes	1.33	0.81–2.20	
Rainy season	Use of bed net	no	1		0.008
		yes	0.6	0.40–0.88	
Use of bed net	Season	dry	1		0.91
		rainy	0.92	0.53–1.61	
No use of bed net	Season	dry	1		< 0.001
		rainy	2.06	1.52–2.79	

### Prevalence of infection

The age of a child, the use of a bed net, equipment and education levels, season, the land tenure of the living area, and the distance of the compound to the drinking fountain were significantly associated with the prevalence of infection in bivariate analysis (Table 2). According to the multivariate random-effect logistic regression model (Table 4), the prevalence of infection was higher among the oldest children (OR = 1.90; 95%CI: 1.58–2.28) and during the rainy season (OR = 2.03; 95%CI: 1.65–2.48). It was lower when the equipment and education indexes were high, OR = 0.53 (95%CI: 0.43–0.66) and OR = 0.75 (95%CI: 0.61–0.92), respectively, and when the children used a bed net (OR = 0.72; 95%CI: 0.57–0.92). Concerning the urban variables, the prevalence of infection was higher in sparsely built-up areas (OR = 1.61; 95%CI: 1.03–2.53) and in irregular areas (OR = 1.85; 95%CI: 1.17–2.92). The observed and expected prevalences in each area were also examined, and appeared quite similar. The area under the ROC curve was 0.82. Gender, the fact that the child had traveled out of Ouagadougou during the preceding month, and the distance of the compound from the hydrographic network or the artificial lakes were not significant variables for explaining the prevalence of infection.

**Table 4:** Multivariate analysis of the prevalence of *Plasmodium falciparum *infection.

		Prevalence of parasite infection		
Variable	Modality	OR	95% CI	p-values
Age	0–6	1		< 0.001
	07–12	1.90	1.58–2.28	
Land tenure	regular	1		0.02
	irregular	1.85	1.17–2.92	
Building density	high	1.00		0.062
	sparse	1.61	1.03–2.53	
Equipment	low	1		< 0.001
	high	0.53	0.43–0.66	
Education level	low	1		0.007
	high	0.75	0.61–0.92	
Use of bed net	no	1		0.008
	yes	0.72	0.57–0.92	
Season	dry	1		< 0.001
	rainy	2.03	1.65–2.48	

### Parasite density in positive children

The age of a child, the season, and travel outside of Ouagadougou during the preceding month were significantly associated with parasite density in bivariate analysis (Table 2). According to the multivariate random-effect linear regression (Table 5), parasite density was lower among the children in the older age group (density multiplied by 0.73) while it was higher when the children had traveled outside of Ouagadougou during the preceding month (density multiplied by 1.90) and during the rainy season (density multiplied by 3.19). Concerning the urban variables, parasite density was not significantly higher in sparsely built-up areas (density multiplied by 1.22), and was not significantly different between regular and irregular areas. Gender, use of bed net, equipment and education levels, and the distance of the compound from the hydrographic network, the drinking fountain or the artificial lakes were not significant variables for explaining parasite density.

**Table 5:** Multivariate analysis of the *Plasmodium falciparum *parasite density among positive children.

		Parasite density (positive children)		
	Modality	Multiplicative factor	95% CI	p-values
Intercept (parasites/μL)		281.5	186–425	
Age	0–6 y.	1		0.02
	7–12 y.	x0.73	0.56–0.94	
Land tenure	regular	1		0.78
	irregular	x1.04	0.76–1.42	
Building density	high	1		0.18
	sparse	x1.22	0.91–1.64	
Travel outside of Ouagadougou the preceding month	no	1		0.02
	yes	x1.90	1.14–3.16	
Season	dry	1		< 0.001
	rainy	x3.19	2.42–4.20	

### Prevalence of malaria attacks

The number of fevers with a parasitaemia > 1,000 μL (definition A) varied from one attack per area (Dapoya, 348 children; Patte d'Oie, 214 children) to seven attacks per area (Somgande, 430 children). The number of fevers with a parasitaemia > 0 (definition B) varied from three attacks per area (Patte d'Oie) to 16 attacks per area (Somgande). There were no significant differences between areas, whatever the clinical case definition. There were three clinical malaria attacks (0.19%) in the dry season and 28 (1.56%) in the rainy season according to definition A (OR = 8.24; 95%CI: 0–86647, p = 0.11), and 18 clinical malaria attacks (1.15%) in the dry season and 50 (2.79%) in the rainy season according to definition B (OR = 2.71; 95%CI: 0.04–177.7, p = 0.15). There was no significant difference in the rate of clinical malaria attacks according to age. No multivariate analysis was performed because the number of attacks was too small.

### Spatial organization

Before taking explanatory variables into account, the p-values of the global Moran tests for the prevalence of infection and parasite density were 0.02 and 0.001, respectively; hence, these variables were spatially correlated. Testing the random-effects of the fitted model, including explanatory variables using the Moran tests, indicated that there was no residual spatial correlation when the explanatory variables were taken into account (p = 0.29 and p = 0.63 respectively). For the prevalence of anti-CSP antibodies, the p-value of the global Moran test was 0.10, so the prevalence of anti-CSP antibodies was not significantly spatially correlated.

## Discussion

Variations in the intensity of malaria transmission can be important in different areas of a town in sub-Saharan Africa [1,9-18]. In Ouagadougou, the prevalence of malaria is heterogeneous and has been found to be related to the spatial and temporal distribution of *Anopheles gambiae s. l. *larval breeding sites [18,25,35,36,42]. For a better knowledge of the local endemicity, the design of the present study was based on the selection of areas characterized by different urbanization processes (regular/irregular land tenure, densely/sparsely built-up areas). Malaria burden was measured by the prevalence and density of infection, the prevalence of clinical malaria and the prevalence of anti-CSP antibodies which can be used as a sero-epidemiological marker of the exposure to malaria transmission [4,37,39,40].

The prevalence and density of infection, and the prevalence of anti-CSP antibodies varied according to the season, as already shown by others in Ouagadougou [25]. Concerning anti-CSP antibody prevalence, an interaction was found between season and the use of a bed net: the use of a bed net was protective only during the rainy season (OR = 0.6; 95%CI: 0.40–0.88), and rainy season, *i.e. *high transmission season, only had an effect (OR = 2.06; 95%CI: 1.52–2.79) when children did not use a bed net. This may be explained by very low vector densities during the dry season and a corresponding lack of contrast in exposure according to bed net use. The use of a bed net was also significantly associated with a lower prevalence of infection (OR = 0.72; 95%CI: 0.57–0.92), as expected [25], without any interaction with season.

Children between seven and 12 years of age had a higher risk of having anti-CSP antibodies or an infection than younger children (OR = 2.60; 95%CI: 2.0–3.35 for anti-CSP antibodies and OR = 1.90; 95%CI: 1.58–2.28 for the prevalence of infection). Young children obviously have less skin surface exposed to mosquito bites and are consequently less exposed to malaria transmission [43]. Moreover, the Ministry of Health recommends that young children (< 5 years) sleep under a bed net and take anti-malarial drugs in case of a fever. A significant association between young age and bed net use (p < 0.001) was found. However, multivariate analysis has shown that the age effect was independent of bed net use. The oldest children have a higher cumulative exposure to malaria transmission and infection, with less frequent use of anti-malaria drugs [4]. Among the younger children, cumulative exposure is lower and malaria treatment more frequent. Parasite density was significantly lower among infected children more than 6 years old (multiplicative factor = 0.73 95%CI: 0.56–0.94). This result could be related to acquired immunity which allows control of high parasitaemia.

Independent of bed net use, higher socio-economic level (according to equipment and education levels) was associated with a significantly lower risk of infection (OR = 0.75; 95%CI: 0.61–0.92 for education level and OR = 0.53; 95%CI: 0.43–0.66 for equipment), without significant associations with the prevalence of anti-CSP antibodies *i.e. *the exposure to malaria transmission. CSP is an antigen that is only expressed at the pre-erythrocytic stages of the parasite. These stages are not sensible to the drugs used in Burkina-Faso (*i.e. *amino-8-quinolines are not used). Therefore, the antibody responses against CSP depend on exposure to malaria transmission but not on anti-malarial treatments (*i.e. *access to drugs and health cares) or resistance of the parasite to these drugs. On the other hand, the prevalence of parasitaemia depends both on malaria transmission, access to anti-malarial treatments and drug efficacy. Higher socioeconomic levels are supposed to be associated with easier access to prevention and treatment as described in other African cities [27,44]. Then, the present results suggest that higher socio-economic status makes easier the access to anti-parasite interventions, *i.e. *anti-malarial treatment, more than to anti-vector measures independent of bed net use.

Even if the risk of having anti-CSP antibodies was not significantly associated with socio-economic variables, it was associated with the two urban variables (land tenure: OR = 1.41; 95%CI: 1.08–1.86 in irregular areas; building density of the area: OR = 1.41; 95%CI: 1.08–1.83 in sparsely built-up areas). The prevalence of infection was also significantly higher in irregular areas (OR = 1.85; 95%CI: 1.17–2.92) and in sparsely built-up areas (OR = 1.61; 95%CI: 1.03–2.53). Then, malaria risk was higher in irregular areas, where its transmission is known to be higher in Ouagadougou [42]. In these irregular areas, the bricks used for constructions are manufactured with soil taken in pits which can be water-filled during the rainy season and then can serve as breeding sites during the dry season. Moreover, some agriculture can exist in irregular urban areas. These urban agriculture areas have been shown to be associated with higher risk of malaria transmission [9,13,25-27]. In contrast, the breeding sites present in regular areas are generally smaller and temporary. Malaria risk was also higher in sparsely built-up areas than in densely built-up ones independent of land tenure. This may be related to the presence of certain vegetation and gardens, puddles, pools, and water bodies that could serve either as resting site for imago [9] or as breeding sites. Moreover, access to a human blood meal is easier for anthropophilic mosquitoes in high population density areas, hence the number of hosts increases and the risk of any single-host receiving an infective bite is reduced [1].

The further children lived away from the hydrographic network (> 500 m), the lower their risk of having anti-CSP antibodies was (OR = 0.66; 95%CI: 0.48–0.90). It is known that the hydrographic network and the artificial lakes are favourable breeding sites for *An. gambiae s.l. *[25,42]. Sabatinelli *et al *[18] showed that in Ouagadougou most of *An. gambiae s.l. *were collected within a distance of 300 meters from the breeding sites. The distance to the artificial lakes was not identified as a risk factor in the present study. Indeed, only 1% of the surveyed households were close to any artificial lake (less than 200 m), and most of the studied areas were several hundreds of meters from artificial lakes (Additional file 1), beyond the flight capacity of *An. gambiae s.l*. in Ouagadougou [18]. Due to a lack of contrasting data, the power of the tests concerning the distance to the artificial lakes was too low to find any significant effect.

Children who traveled out of Ouagadougou during the preceding month had a higher risk for having anti-CSP antibodies (OR = 1.91; 95%CI: 1.26–2.90) and higher parasite densities (multiplicative factor = 1.90; 95%CI: 1.14–3.16). Robert *et al *[1] reported that nearly everywhere in sub-Saharan Africa anopheline species density and the likelihood of malaria transmission are higher in rural areas than urban. About 7% of the surveyed children traveled out of Ouagadougou during the month preceding the study. In addition to urban transmission, exposure to rural transmission is a significant source of infection for children living in Ouagadougou. Moreover, the exposure to rural transmission and the import of parasites from surrounding areas to the cities contribute to the genetic diversity of *P. falciparum *populations found in urban areas [45].

A difference between malaria attacks in different areas or age groups could not been shown due to insufficient statistical power for a multivariate analysis. However, a difference in the number of attacks between the dry and the rainy season (OR = 8.24 for definition 1, OR = 2.71 for definition 2) was found, as expected, and as reported by others [20,25].

The prevalence of infection and parasite density were spatially correlated before taking the explanative variables into account. According to the Moran test, these spatial correlations could be explained by the variables included in the mixed models. A similar lack of spatial correlation has also been shown in another study of urban malaria [27]. Moreover, according to the area under the ROC curve, the logistic models for the prevalence of anti-CSP antibodies and for the prevalence of infection had sufficient capacity to discriminate among children with or without anti-CSP antibodies and with or without infection.

The results of the present study are similar to those of Wang *et al *who conducted a rapid malaria appraisal in Ouagadougou in 2002, two years before the present study, and published their results in 2005 [25]. They also found a peak in the prevalence of malaria infection among older children, those not using a bed net, and those living near agricultural land or a garden. They did not find the prevalence of infection to be associated with travel outside of Ouagadougou while they found an association between the malaria infection rates and travels in rural areas around other African cities: Dar El Salaam and Abidjan [22,24]. It is interesting to note that in the present study this risk factor was associated with the prevalence of anti-CSP antibodies (not influenced by the use of medicine) and parasite density, not with the prevalence of infection (as in the study conducted by Wang *et al*).

Wang *et al *[25] found an infection rate of 24.1% for school children living in the centre of Ouagadougou and 68.7% for children living in the periphery at the end of November 2002. These prevalence rates are higher than those observed in the present study: 9.3%, 11.6% and 12.4% in Patte d'Oie, Gounghin and Dapoya, respectively (in the centre), to 32.3% in Zongo (in the periphery). The difference could be related to the younger age of children (between 6 months and 12 years old in the present study compared to between 6 and 10 years in the studies of Wang *et al*), a different period of recruitment or lower reading effort for the blood films (up to 200 leukocytes in the present study compared to 500 leukocytes in the studies of Wang *et al*).

In conclusion, the present results contribute to a better understanding of malaria epidemiology in Ouagadougou and confirm the results of previous studies [25,35,36]. Urban malaria transmission is low with a marked seasonality and malaria immunity acquisition is consequently delayed. The transmission sites are mainly situated in irregular and sparsely built-up areas and within 200–300 meters of the hydrographic system. Poorer households are at greater risk of infection independently of bed net use. Finally, traveling outside of Ouagadougou increases the risk of being exposed to malaria transmission and high parasitaemia. Therefore, to reduce malaria burden in Ouagadougou, it may be necessary to work in irregular and sparsely built-up areas. Areas near the hydrographic network should be the principal targets. All these at-risk areas could easily be identified and monitored using remote sensing. It could be also useful to take into consideration the malaria infections imported from surroundings rural areas by urban city-dwellers having low acquired immunity. Urban children from Ouagadougou would benefit from preventive interventions (e.g. anti-vectorial devices or chemoprophylaxis) aimed at reducing malaria risk during and after their travels to rural areas.

## Competing interests

The authors declare that they have no competing interests.

## Authors' contributions

MB carried out statistical analysis and interpretation of the data, drafted and revised the manuscript. FF was the principal scientific investigator of the study and revised the manuscript. MCH was the investigator responsible of the malaria part of the study, participated in the study design, coordinated the malaria field and laboratory work in Burkina Faso, interpreted the data and revised the manuscript. SA conducted immunological work in the research department of CR. CR participated in the design of the malaria part of the study, supervised the immunological and statistical analysis, interpreted the data, drafted and revised the manuscript. HO took part in the field work and study design. GS conceived the research programme. All authors read and approved the final manuscript. None declared conflict of interest.

## Supplementary Material

Additional file 1 **Description of quantitative independent variables in the areas**. The data provided for each area are the number of compounds, the number of households and the number of children aged 6 months-12 years that have been investigated. It presents also the age of children (median, 25% percentile and 75% percentile), the distances from the compounds to the closest hydrographic network, to the closest drinking fountain and to the closest artificial lake (median, 25% percentile and 75% percentile). Click here for file
