# Supplementary material for: Social and environmental malaria risk factors in urban areas of Ouagadougou, Burkina Faso
Source: Malar J. 2009 Jan 13;8:13. doi: 10.1186/1475-2875-8-13 (PMC2640411; doi:10.1186/1475-2875-8-13)
Supplement: Additional file 1 — Description of quantitative independent variables in the areas. The data provided for each area are the number of compounds, the number of households and the number of children aged 6 months-12 years that have been investigated. It presents also the age of children (median, 25% percentile and 75% percentile), the distances from the compounds to the closest hydrographic network, to the closest drinking fountain and to the closest artificial lake (median, 25% percentile and 75% percentile). [file 1475-2875-8-13-S1.doc]

**Additional file 1 : Description of quantitative independent variables in the areas**

| Land tenure |  | Irregular | | | | Regular | | | |
| --- | --- | --- | --- | --- | --- | --- | --- | --- | --- |
| Building density |  | Sparse | | Dense | | Sparse | | Dense | |
| Area |  | Burundi | Zongo | Somgandé | Yamtenga | Gounghin | Tanghin | Dapoya | Patte d’oie |
| Variable |  |  |  |  |  |  |  |  |  |
| No. compounds |  | 188 | 194 | 176 | 223 | 132 | 170 | 103 | 82 |
| No. households |  | 194 | 194 | 178 | 223 | 148 | 172 | 128 | 86 |
| No. of children aged 6 months-12 years |  | 497 | 474 | 430 | 549 | 397 | 445 | 348 | 214 |
| Age (years) | Q25* | 3 | 3 | 3 | 3 | 4 | 4 | 3 | 4 |
|  | median | 6 | 5 | 6 | 6 | 7 | 7 | 6 | 7 |
|  | Q75** | 10 | 9 | 9 | 9 | 10 | 10 | 9 | 10 |
| Distance from the compounds to the closest hydrographic network (meters) | Q25* | 309.6 | 82.3 | 99.92 | 556.4 | 202.8 | 480 | 203.5 | 116.8 |
|  | median | 620.1 | 131.4 | 179.4 | 719.5 | 345.3 | 774.6 | 395.7 | 276.1 |
|  | Q75** | 829.9 | 258.6 | 252.1 | 885.2 | 458.8 | 1061 | 597.2 | 356.7 |
| Distance from the compounds to the closest fountain (meters) | Q25* | 143.7 | 470 | 202.4 | 165.5 | 179.9 | 100.6 | 86.16 | 226.5 |
|  | median | 255.2 | 612.4 | 256.6 | 278.8 | 242.8 | 143.5 | 118.7 | 309.3 |
|  | Q75** | 375.1 | 774.4 | 326.6 | 427.8 | 317.9 | 178.7 | 183.3 | 398.6 |
| Distance from the compounds to the closest artificial lake (meters) | Q25* | 2160 | 1421 | 2949 | 1823 | 3550 | 575.6 | 347.5 | 2067 |
|  | median | 2370 | 1730 | 3062 | 1948 | 3667 | 1048 | 550 | 2315 |
|  | Q75** | 2568 | 1902 | 3209 | 2048 | 3786 | 1402 | 698.1 | 2562 |

*Q25: 25% percentile, **Q75: 75% percentile.
